# Assessment of Stress, Depressive and Anxiety Symptoms in Patients with COPD during In-Hospital Pulmonary Rehabilitation: An Observational Cohort Study

**DOI:** 10.3390/medicina57030197

**Published:** 2021-02-25

**Authors:** Adam Wrzeciono, Oliver Czech, Katarzyna Buchta, Sabina Zabłotni, Edyta Gos, Łukasz Tłuczykont, Dagmara Górecka, Agnieszka Pastuła, Mateusz Adamczyk, Ewa Jach, Igor Świerkowski, Patryk Szary, Jan Szczegielniak

**Affiliations:** 1 Descartes’ Error Student Research Association, Department of Physical Education and Physiotherapy, Opole University of Technology, 45-758 Opole, Poland; oliver.czech@student.po.edu.pl (O.C.); k.buchta@student.po.edu.pl (K.B.); s.zablotni@student.po.edu.pl (S.Z.); e.gos@student.po.edu.pl (E.G.); lukasz.tluczykont@student.po.edu.pl (Ł.T.); d.gorecka8223@student.po.edu.pl (D.G.); agnieszka.pastula@student.po.edu.pl (A.P.); ma.adamczyk@student.po.edu.pl (M.A.); ewa.jach@student.po.edu.pl (E.J.); swierkowski.eu@gmail.com (I.Ś.); 2 Faculty of Physiotherapy, University School of Physical Education in Wroclaw, 51-612 Wroclaw, Poland; p-szary@o2.pl; 3 Department of Physical Education and Physiotherapy, Opole University of Technology, 45-758 Opole, Poland; j.szczegielniak@po.edu.pl

**Keywords:** pulmonary rehabilitation, respiratory disease, behavioral symptoms, emotions

## Abstract

*Background and Objectives*: The relationship between physical health and mental health has been considered for years. A number of studies have shown a correlation between depressive states and the progress of somatic diseases. It seems that the proper cooperation of specialists may result in the improvement of the patient’s well-being and a positive effect on the course of the rehabilitation process. The aim of this study was to assess the symptoms of depression, anxiety, and stress in patients with chronic obstructive pulmonary disease (COPD) as well as the assessment of the relationship of psychological symptoms with sociodemographic factors and physical condition. *Materials and Methods*: The study enrolled 51 COPD patients who underwent a three-week pulmonary rehabilitation program. After admission to the rehabilitation department, the subjects were asked to complete the Hospital Anxiety and Depression Scale (HADS) questionnaire, the Perception of Stress Questionnaire (PSQ), and a sociodemographic questionnaire. *Results*: Anxiety states were diagnosed in 70% of respondents and depressive states were diagnosed in 54% of patients. Some of the respondents (14%) also showed a tendency to experience various grounded stresses. Additionally, there were correlations between the mental state and the results of fitness and respiratory tests. *Conclusions*: Patients with COPD are at risk for mental disorders, which may adversely affect their general health and significantly limit their physical and respiratory efficiencies. The development of widely available therapeutic solutions to reduce symptoms associated with depression, anxiety, and stress seems to be an important challenge for the management of patients with COPD.

## 1. Introduction

Chronic obstructive pulmonary disease (COPD), manifested by coughing, shortness of breath, sputum production, wheezing, and reduced physical performance, is one of the most frequently diagnosed lung diseases [1]. However, the symptoms are not just respiratory, because numerous disorders, indirectly related to respiratory disorders, are also noticed in COPD patients [2]. The number of cases is systematically growing, and the World Health Organization (WHO) predicts that by 2030 COPD may become the third leading cause of death worldwide [3].

It is estimated that one in four people with COPD has symptoms of depression and anxiety. It has been proven that the more advanced the disease is, the greater the risk of depressive symptoms [4], with women being more prone to depression [5]. Moreover, previous studies have shown that people with depression use cigarettes more often, and the attempt to quit smoking causes depressive symptoms. On the other hand, continuing to smoke tobacco products leads to the development of the disease and reduces the quality of life [6]. The relationship between health and depression is confirmed by Torrisi et al. The study has shown that patients presenting with milder depressive states have a better quality of life [7].

Depressive states, which are common among patients with COPD, influence the course of pulmonary rehabilitation. A reduction of depressive symptoms makes pulmonary rehabilitation more effective, and patients are more motivated to cooperate actively with the therapist in the rehabilitation process [8]. An interdisciplinary therapeutic team can holistically take care of the patient and thus plan a therapy focused on all the biopsychosocial problems of the patient [9].

The study aimed to assess the symptoms of depression, anxiety, and stress in patients with COPD participating in an in-hospital pulmonary rehabilitation program, as well as the evaluation of the relationship between psychological symptoms with sociodemographic factors and physical condition. The hypotheses were as follows: (H1) Patients with COPD present symptoms of depression and anxiety and stress; (H2) professional activity will correlate with perceived stress and symptoms of anxiety and depression; (H3) cigarette smoking will correlate with perceived stress as well as symptoms of anxiety and depression; (H4) the undertaking of voluntary physical activity by patients will correlate with the lower occurrence of stress, anxiety, and depression symptoms.

## 2. Materials and Methods

### 2.1. Participants

The study was conducted among patients who participated in an inpatients pulmonary rehabilitation at the Specialist Hospital in Glucholazy (Poland) in October 2020. The research enrolled 51 randomly selected patients aged 48–84 years old who met the inclusion criteria and gave written consent to participate in the study. The main group characteristics are presented in Table 1. Inclusion criteria were: women and men aged 45–85, COPD as the main diagnosis regardless of the disease phase, both smokers, non-smokers and ex-smokers, no diagnosed psychological disorders as well as a written consent to participate in the study. The exclusion criteria were: age below 45 and above 85, the main diagnosis other than COPD, presence of consciousness disturbances, inability to independently complete the research questionnaires or the refusal of consent from the patient. The study was designed according to the Strobe guidelines for cohort studies, approved by the Research Ethics Committee of the University School of Physical Education (Resolution No. 18/2020), registered in ClinicalTrials.gov (accessed on 1 October 2020) (NCT04726917), and carried out in accordance with the Declaration of Helsinki guidelines.

### 2.2. Measurement

At the beginning, the participants completed the proprietary sociodemographic questionnaire. The questions concerned basic personal data, physical activity, stimulants, or perception of one’s own health condition. The collected data was used for group characteristics and statistical analysis.

#### 2.2.1. Hospital Anxiety and Depression Scale

The Polish version of the Hospital Anxiety and Depression Scale (HADS) was used to assess depression and anxiety in patients. The questionnaire is considered a reliable method of assessing anxiety and depression [10]. The HADS consists of 14 questions that are rated on a 4-point (0 to 3) scale, with 3 representing the highest level of anxiety or depression. In the questionnaire, there are two separate subscales that give partial scores: HADS-A for anxiety and HADS-D for depression. Obtaining 8 out of 21 points in each subscale is considered a cut-off point, which means that patients who score 8 or more points on at least one subscale are classified in the group characterized by a varying degree of anxiety or depressive disorders. Values 0–7 are taken as the norm, and values 8–10 indicate mild anxiety or depressive disorder. It is assumed that results in the range 11–21 indicate severe anxiety or depressive disorder. A literature review based on 747 articles showed that reaching a minimum score of 8 achieves the highest specificity between the sensitivity and specificity of the test. The HADS-A had optimal cut off ≥ 8 (sensitivity 0.89, specificity 0.75) [11]. The Cronbach’s alpha for HADS-A was α = 0.83, and for HADS-D was α = 0.82 [12].

#### 2.2.2. Perception of Stress Questionnaire

The Polish Kwestionariusz Poczucia Stresu—in English translation: The Perception of Stress Questionnaire (PSQ)—by Plopa and Makarowski consists of 27 questions that allow determining the level of external stress, emotional tension, and intrapsychic stress, in addition to the risk of lying. Using a 5-point Likert scale (true, rather true, hard to say, rather not true, or untrue), patients will determine the given problem degree. Interpreting the overall score between 21 and 105, with a cut-off point of 60, allows determining the patient’s stress level. The questionnaire authors obtained reliability indicators at the level of: for external stress α = 0.72, for emotional tension α = 0.81 and for intrapsychic stress α = 0.69 [13].

#### 2.2.3. Initial Assessment of Patients

The pulmonary rehabilitation was carried out in five subgroups depending on the functional states of the patients. Factors such as coexisting cardiovascular diseases, age, fatigue level, and shortness of breath after the test were taken into consideration. The 6-min walk test (6MWT) and spirometry were the basis for the qualification process of patients.

During the 6MWT, the patient’s endurance and aerobic capacity was assessed [14]. The patient’s task was to cover as much distance as possible walking as quickly as possible without stopping for 6 min. The assessed parameters were distance traveled, energy expenditure (expressed in MET), number of breaks and their duration, and the levels of dyspnea and fatigue using a 10-point Modified Borg Scale [15]. The body’s hemodynamic response to exercise was also measured. The results of 6MWT were expressed as metabolic equivalent (MET) calculated based on the following formula [16]:

MET = −0.0971 × V3 +1.5021 × V2 − 5.3762 × V.

Spirometry is a basic test of respiratory function. Lung capacity and airflow through the lungs and bronchi are the assessed parameters. The main purposes of spirometry are to monitor the course of the disease and the effects of therapy. The parameter used to qualify patients for pulmonary rehabilitation is forced expiratory volume in one second (FEV1). Spirometry was performed using the Jaeger MasterLab (Erich-Jaeger) device.

After the initial assessment and baseline measures, patients participated in a two-week, traditional pulmonary rehabilitation. After completion of rehabilitation, the patients underwent final assessment consisting of 6MWT and spirometry to evaluate functional improvement as well as stress, anxiety, and depression follow-up evaluation. However, the results of final measurements and improvement will be a part of another publication.

### 2.3. Statistical Analysis

All statistical analyses were performed using Statistica 13 software (StatSoft, Cracow, Poland). The statistical significance level was set at α = 0.05. Categorical variables were presented as numeric values and percentages. Continuous variables were presented as mean ± standard deviation (SD). The correlation analysis was performed using R rang Spearman’s test.

## 3. Results

### 3.1. Characteristics of the Group

The analyzed data were obtained from 51 patients. All participants suffered from COPD. Among the patients, 9 (18%) had primary or vocational education, 13 (26%) had higher education, and 28 (56%) had secondary education. Only 11 respondents (22%) were still professionally active, and 22 respondents (78%) were receiving a retirement pension or a sickness pension; 26 (52%) of the study participants were married, 22 (44%) were widowed or divorced, and 2 were single. In addition, 31 patients (61%) suffered from at least two comorbidities (e.g., hypertension, as presented by 22 patients (44%)).

Additionally, the participants determined the level of undertaken physical activity. A total of 28 patients (56%) declared that they regularly undertook physical activity (e.g., walking, cycling, or swimming); 21 patients (41%) undertook physical activity occasionally, and 2 patients (4%) did not undertake any additional physical activity. Patients were also asked about smoking, now and in the past. Six participants (12%) declared that they continued to smoke, and 24 participants (48%) had smoked cigarettes in the past. When asked about the self-assessment of their health condition, only 10 patients (20%) assessed their health as good, 8 patients (16%) indicated that their health was bad and the others assessed their health as average. The sociodemographic characteristic is presented in Table 2.

### 3.2. Assessment of Depression, Anxiety, and Stress Symptoms

The analysis of the results showed that the mean score in the study group for depression (HADS-D) was 7.3 (±2.8) points. In all, 27 patients (54%) exceeded the cut-off point (score ≥ 8 points) and were prone to depressive disorders. The mean score for anxiety (HADS-A) was 9.4 (±3.5) points and 35 patients (70%), exceeding the cut-off point (score ≥ 8 points), were prone to anxiety.

The analysis of the PSQ results showed that the patients’ mean scores were for emotional tension 23.6 (±6.0) points, for external stress 17.8 (±5.9) points, and for intrapsychic stress 19.0 (±6.4) points. The analysis of the overall PSQ score showed that seven participants (14%) achieved at least 80 points and thus showed a tendency to feel stress. The clinical characteristics are presented in Table 1.

### 3.3. Correlation Analysis

The mental state data of the patients, as well as the results of the (6MWT) and spirometry obtained during qualification for rehabilitation, were statistically analyzed for the presence of correlations. The analysis of the results showed a number of statistically significant positive and negative correlations (Table 3).

Significantly positive correlations were found between the HADS-A score for anxiety evaluation, the score of emotional tension (*R* = 0.61), the score of external stress (*R* = 0.50), the level of intrapsychic stress (*R* = 0.69), and the overall PSQ score (*R* = 0.70). Such a correlation could be interpreted as: the more the patient is prone to stress, the higher the risk of anxiety. The HADS-A score also correlates with smoking (*R* = −29). It may mean that patients who smoke are less prone to anxiety. The score of intrapsychic stress is also related to the professional activity (*R* = 0.32) and the level of physical activity (*R* = 0.32). It means that patients who are professionally active or rarely engage in physical activity experience stress more often. Age and sex did not show any significant correlation with the measured parameters.

#### Correlations with the Results of the Spirometry Test and the 6MWT

The perceived stress significantly affected the results of the spirometry test. The lower result of the forced expiratory volume in one second is related to the score of perceived general stress (*R* = −0.38), the score of external stress (*R* = −0.43), and the score of intrapsychic stress (*R* = −0.53). Such correlations mean that the more the patients are prone to perceived stress, the less efficient is the respiratory function. It was also shown that the patients who occasionally or never engage in recreational physical activity had a significantly worse FEV1% result (*R* = −0.31). Age and sex did not show any significant correlation with the measured parameters.

Many factors affect the 6MWT results and, therefore, the patient’s exercise tolerance. The data analysis confirmed that the 6MWT results significantly decreased with extinction of professional activity (*R* = −0.38) and decreasing physical activity (*R* = −0.36). Supplementary Materials Table S1 presents correlations of all analyzed factors.

## 4. Discussion

COPD, as a chronic disease is characterized by many symptoms that also affect other systems besides the respiratory system. Systemic somatic alterations influence changes in patients’ psyches and vice versa. It is assumed that dyspnea accompanying COPD leads to a reduction in the quality of life, dyspnea-related anxiety, and fear in patients with COPD [17]. Moreover, dyspnea and related fatigue may lead to respiratory distress accompanied by reduced exercise capacity. Evidence also suggests that fear affects the avoidance of physical activity [17,18]. In this group of patients, the severity of the disease is reflected in progressive sadness, which can eventually lead to depression [19]. Research also suggests that symptoms of dyspnea often lead to anxiety. Moreover, anxiety and depression can exacerbate dyspnea symptoms. This leads to a vicious circle formation in which dyspnea causes an increase in the level of anxiety and depression, and vice versa—psychological symptoms intensify dyspnea [20,21,22]. Although, pulmonary rehabilitation programs can lead to a reduction in dyspnea, which in turn can reduce symptoms of stress or anxiety [23]. The proven impact of anxiety associated with dyspnea on increasing the disability degree has made the assessment and treatment of anxiety an integral part of clinical practice in the treatment of COPD [24]. The genetic determinants of depression in the course of COPD are also being sought. A study by Rybka et al. demonstrated that depressive symptoms during COPD may be related to increased levels of interleukin 6 (IL-6) [25]. Interestingly, elevated IL-6 levels are observed in COPD as well as in depression itself.

The aim of this study was to assess the occurrence of depression, anxiety, and stress symptoms in patients with COPD, as well as the assessment of the relationship of psychological symptoms with sociodemographic factors and physical condition. Our first hypothesis assumed that patients with COPD are prone to perceive stress, depression, and anxiety symptoms. Analysis of the results confirmed the hypothesis, showing an occurrence of stress, anxiety, and depression symptoms. Fifty-three percent of patients with the mean score in HADS-D 7.3 (±2.8) are prone to depressive disorders and 70% with the mean score in HADS-A 9.4 (±3.5) are prone to anxiety. It seems that this may be related to the early stages of the disease. Patients are unaware of how the disease can change their lives, so they are more prone to stress and anxiety. The meta-analysis of Zhang et al. showed that in a stable COPD, 10–42% of patients are prone to depression and 10–19% are prone to anxiety. In severe COPD, the risk of depression significantly increases—up to 62% [22]. The incidence of depression and anxiety is also high in patients who have recently experienced an exacerbation in COPD. Depression occurs in 19–50% of patients, while anxiety in 9–58% [26]. The results of our study indicate higher values than in similar studies. Puhan et al. examined patients with moderate COPD. The mean score for depression in HADS was 7.63 (±3.9) and 21.6% of patients had a probable clinical diagnosis of depression scoring ≥ 11 in HADS-D, while the mean score for anxiety was 7.03 (±4.0) and 22.7% of patients were likely to be clinically diagnosed with anxiety, scoring ≥ 11 in HADS-A [27]. Smid et al. investigated the anxiety and depression levels in patients with mild to very severe COPD using HADS. The results show that the mean score for depression was 7.4 (±4.2) and for anxiety 7.5 (±4.4) [28]. Dowson et al. indicted that depression affects 28%, and anxiety 50% of COPD patients. Moreover, in this study, the level of depression and anxiety were correlated with the severity of the disease. The more severe the COPD, the higher the depression score and the anxiety score [29]. An analysis of the results in our study showed that 14% of patients tend to feel stress. This is in line with a study by Yohannes et al., which showed that 25% of COPD patients are prone to feel stressed (mild stress-8%, moderate-8%, severe-6%, extremely severe-3%) [30]. These results indicate that depression and anxiety often accompany COPD patients, regardless of the stage of the disease. The different frequency of symptoms and their varying severity emphasize the importance of diagnosis and the need to treat mental disorders among patients with COPD.

The second hypothesis assumed that professional activity increases the tendency to perceive stress. Analysis of the results confirmed that professional activity is strongly associated with the perception of intrapsychic stress. Professionally active persons experience significantly higher scores of intrapsychic stress than retirees. The meta-analysis of Madsen et al. indicates that depression among employees may be caused by strain associated with their job [31]. Therefore, it seems important to reduce psychological symptoms, especially in patients with COPD who remain professionally active. Such actions can inhibit the rapid progression of the disease and improve patients’ quality of life

The third hypothesis assumed a correlation between smoking and perceived stress as well as symptoms of anxiety and depression. Analysis of the results confirms this hypothesis; however, an inverse correlation was noted between active cigarette smoking and anxiety symptoms, indicating that cigarette smokers presented anxiety symptoms less often. The literature confirms that in the course of COPD, smoking leads to the development of the disease and reduces quality of life [6], but in the present study there was no relationship.

The fourth hypothesis assumed that voluntary physical activity will correlate with a lower occurrence of stress, anxiety, and depression symptoms. The analysis of the results did not confirm this hypothesis. Although, there may be bias in the reported PA.

Many studies have shown that exercise has a positive effect on improving mental health in COPD patients. Physical rehabilitation, regardless of duration, significantly reduces the symptoms of anxiety and depression [32,33,34]. On the other hand, Lin et al. and Valenza et al. clearly showed that controlled breathing training, which is especially recommended for pulmonary patients, significantly alleviates the symptoms of depression and anxiety in patients with COPD [35,36]. Psychological symptoms could also reduce the effectiveness of the pulmonary rehabilitation process. Cullen et al. demonstrated that symptoms of depression and anxiety in COPD patients lower exercise tolerance and increase the progression of disease [37]. Therefore, alleviating the symptoms of stress, anxiety, and depression may be important in increasing the effectiveness of rehabilitation. A number of studies have assessed the impact of psychological interventions on the severity of symptoms of anxiety and depression. Luk et al. and Coventry et al. showed that the use of cognitive-behavioral therapy and relaxation techniques in pulmonary patients has a positive effect on their mental state and significantly reduces the symptoms of depression and anxiety in patients with COPD [38,39]. The results of the cited studies clearly show that physical training or psychological therapy resulted in the improvement of the mental state of patients, as well as their fitness and respiratory parameters. In a review, Pumar et al. indicated the importance of comprehensive pulmonary rehabilitation supported by a cognitive-behavioral approach [2]. Lunn et al. indicated the role of psychotherapists in pulmonary rehabilitation. Psychological care in pulmonary diseases is a very important aspect. However, the presence of a psychotherapist in an interdisciplinary therapeutic team is very often overlooked. It may be related to a lack of sufficient funds as well as a small number of qualified behavioral therapy specialists [40]. Therefore, an easy-to-use and effective psychotherapy tool which can be operated, for example, by a physiotherapist is needed.

The topic of this special issue is the management of patients with COPD during COVID-19. In our opinion, such a solution can be the use of new technologies that can be applied by a physiotherapist, such as using virtual reality. In recent years, a growing interest in supplementing standard rehabilitation with virtual reality (VR) therapy, and a wide application of VR in various areas of rehabilitation is being observed [41,42,43]. The results of research suggest that the use of VR-based therapy may positively affect the well-being of patients [44]. Cieslik et al. indicated the positive effect of VR therapy as supportive therapy in patients with psychiatric disorders [45]. Therefore, VR conditions can be an interesting solution to the problems of providing patients with appropriate psychological care. Devices can be easy to use, and thus the specialist presence will not be required during VR psychotherapy. The literature review shows that VR therapy may have a positive effect on patients with behavioral or psychological disorders [46]. Due to the constantly developing technology of VR, an interesting tool could be applications that enable comprehensive rehabilitation of pulmonary patients by combining exercise therapy with behavioral therapy in a VR environment. The use of VR to conduct physical training in pulmonary patients has already been described [47,48,49]. Extending traditional pulmonary rehabilitation with VR training may effectively improve the physical fitness of patients. Rutkowski et al. investigated the effectiveness of using immersive virtual therapy, in comparison to Schultz autogenic training, in reducing the level of stress, anxiety, and depression in patients with COPD. The result shows that VR therapy is a significantly more effective tool in reducing anxiety and depression symptoms in COPD patients than traditional therapy [50]. The most recent pulmonary rehabilitation recommendations indicate the need to modify the behavior of patients in order to increase the level of physical activity. 

The present study has several limitations. Firstly, the study group was small. However, the number of patients in our study was determined by the hospital where the study was conducted. Secondly, the lack of full spirometry examination data in the correlation analysis. Finally, to evaluate patients more broadly, a follow-up evaluation should be performed in future studies.

## 5. Conclusions

Patients with COPD are prone to anxiety, depression, and stress, which may directly affect their general health. Improvement in mental state may increase the effectiveness of pulmonary rehabilitation and inhibit the development of the disease. Due to the lack of qualified behavioral therapists, scientists should focus their attention on alternative methods of psychotherapy that could become an effective tool in relieving symptoms related to depression, anxiety, and stress.

## Figures and Tables

**Table 1:** Group characteristics.

Variables	Mean (SD)
Age (years)	66.0 (7.8)
BMI (kg/m^2^)	28.4 (4.8)
Female, *n* (%)	42 (84%)
FEV1 (%)	78.2 (23.6)
6MWT (MET)	6.06 (1.97)
**HADS**	
Depression assessment (HADS-D)	7.3 (2.8)
Anxiety assessment (HADS-A)	9.4 (3.5)
**PSQ**	
Emotional tension	23.6 (6.0)
External stress	17.8 (5.9)
Intrapsychic stress	19.0 (6.4)
Total score	59.2 (17.8)

**Notes**: FEV1%: forced expiratory volume for 1 s, 6MWT: the 6-min walk test, SD: standard deviation, BMI: Body Mass Index, MET: metabolic equivalent, HADS: Hospital Anxiety and Depression Scale, PSQ: The Perception of Stress Questionnaire.

**Table 2:** Group sociodemographic characteristics.

Variables	*n* (%)
**Education**	
Primary/vocational education	9 (18%)
Secondary education	28 (55%)
Higher education	14 (27%)
**Employment status**	
Professionally active	11 (22%)
Retired	40 (78%)
**Material status**	
Married/cohabiting	27 (53%)
Divorced/widowed	22 (43%)
Single	2 (4%)
**Comorbidities**	
Current	31 (61%)
No comorbidities	20 (39%)
**Voluntary physical activity**	
Regular	28 (55%)
Occasional	21 (41%)
None	2 (4%)
**Smoking**	30 (59%)
Present	6 (12%)
In the past	24 (47%)
**Subjective health status judgement**	
Good	10 (20%)
Average	33 (65%)
Bad	8 (15%)

**Table 3:** Important correlations.

Feature	Professional Activity	Physical Activity	Cigarettes (Currently)	Emotional Tension	External Stress	Intrapsychic Stress	Stress Total Score	6MWT MET	FEV1%	HADS-A
Professional activity	-	0.10	−0.14	0.15	0.17	0.32 *	0.24	−0.38 *	−0.08	0.28
Physical activity	0.10	-	−0.19	0.14	0.17	0.32 *	0.24	−0.36 *	−0.31 *	0.22
Cigarettes (currently)	−0.14	−0.19	-	−0.17	0.07	−0.14	−0.09	0.08	−0.12	−0.29 *
Emotional tension	0.15	0.14	−0.17	-	0.51	0.69	0.85	−0.11	−0.09	0.61 *
External stress	0.17	0.17	0.07	0.51	-	0.68	0.84	−0.07	−0.43 *	0.50 *
Intrapsychic stress	0.32 *	0.32 *	−0.14	0.69	0.68	-	0.90	−0.15	−0.53 *	0.69 *
Stress (total score)	0.24	0.24	−0.09	0.85	0.84	0.90	-	−0.11	−0.38 *	0.70 *
6MWT (MET)	−0.38 *	−0.36 *	0.08	−0.11	−0.07	−0.15	−0.11	-	0.13	−0.10
FEV1%	−0.08	−0.31 *	−0.12	−0.09	−0.43 *	−0.53 *	−0.38 *	0.13	-	−0.22
HADS-A	0.28	0.22	−0.29 *	0.61 *	0.50 *	0.69 *	0.70 *	−0.10	−0.22	-

**Notes**: *: statistically significant correlation, 6MWT: the 6-min walk test, FEV1%: forced expiratory volume for 1 s, MET: metabolic equivalent, HADS-A: Hospital Anxiety and Depression Scale for anxiety assessment.

## Data Availability

The data presented in this study are available on request from the corresponding author.

## Supplementary Materials

The following are available online at https://www.mdpi.com/1648-9144/57/3/197/s1. Table S1: Correlation analysis.
