# Supplementary material for: Assessment of Stress, Depressive and Anxiety Symptoms in Patients with COPD during In-Hospital Pulmonary Rehabilitation: An Observational Cohort Study
Source: Medicina (Kaunas). 2021 Feb 25;57(3):197. doi: 10.3390/medicina57030197 (PMC7996584; doi:10.3390/medicina57030197)
Supplement: Supplementary file 1 [file medicina-57-00197-s001.pdf]

Table S1. Correlation analysis

Feature	Age	BMI	Professional activity	Hypertension	Physical activity	Cigarettes (currently)	Cigarettes (formerly)	Cigarettes (how long)	Emotional tension	External stress	Intrapsychic stress	Stress (overall score)	6MWT (MET)	FEV1%	HADS- D	HADS -A	HADS (A+D)
Age	-	-0.18	0.48	**0.33***	0.08	-0.09	0.12	0.13	0.15	0.01	0.16	0.12	**-0.38***	0.20	0.06	0.03	0.06
BMI	-0.18	-	0.02	**0.39***	0.25	-0.13	-0.24	-0.21	0.16	0.08	0.10	0.12	-0.16	-0.05	-0.01	0.23	0.12
Professional activity	0.48	0.02	-	**0.33***	0.10	-0.14	-0.01	0.00	0.15	0.17	**0.32***	0.24	**-0.38***	-0.08	0.09	0.28	0.23
Hypertension	**0.33***	**0.39***	**0.33***	-	**0.31***	-0.28	-0.21	-0.15	0.21	0.15	**0.30***	0.25	**-0.41***	-0.13	0.23	**0.40***	**0.43***
Physical activity	0.08	0.25	0.10	**0.31***	-	-0.19	0.03	0.00	0.14	0.17	**0.32***	0.24	**-0.36***	**-0.31***	0.27	0.22	**0.32***
Cigarettes (currently)	-0.09	-0.13	-0.14	-0.28	-0.19	-	**0.36***	**0.54***	-0.17	0.07	-0.14	-0.09	0.08	-0.12	-0.12	**-0.29***	-0.25
Cigarettes (formerly)	0.12	-0.24	-0.01	-0.21	0.03	**0.36***	-	**0.88***	-0.22	0.02	0.00	-0.06	0.10	0.05	0.15	-0.17	-0.10
Cigarettes (how long)	0.13	-0.21	0.00	-0.15	0.00	**0.54***	**0.88***	-	-0.11	-0.05	0.03	-0.04	0.09	-0.08	0.15	-0.15	-0.06
Emotional tension	0.15	0.16	0.15	0.21	0.14	-0.17	-0.22	-0.11	-	0.51	0.69	0.85	-0.11	-0.09	0.17	**0.61***	**0.50***
External stress	0.01	0.08	0.17	0.15	0.17	0.07	0.02	-0.05	0.51	-	0.68	0.84	-0.07	**-0.43***	0.16	**0.50***	**0.42***
Intrapsychic stress	0.16	0.10	**0.32***	**0.30***	**0.32***	-0.14	0.00	0.03	0.69	0.68	-	0.90	-0.15	**-0.53***	0.19	**0.69***	**0.54***
Stress (overall score)	0.12	0.12	0.24	0.25	0.24	-0.09	-0.06	-0.04	0.85	0.84	0.90	-	-0.11	**-0.38***	0.21	**0.70***	**0.57***
6MWT (MET)	**-0.38***	-0.16	**-0.38***	**-0.41***	**-0.36***	0.08	0.10	0.09	-0.11	-0.07	-0.15	-0.11	-	0.13	-0.21	-0.10	-0.20
FEV1%	0.20	-0.05	-0.08	-0.13	**-0.31***	-0.12	0.05	-0.08	-0.09	**-0.43***	**-0.53***	**-0.38***	0.13	-	-0.18	-0.22	-0.25
HADS-D	0.06	-0.01	0.09	0.23	0.27	-0.12	0.15	0.15	0.17	0.16	0.19	0.21	-0.21	-0.18	-	0.12	0.70
HADS-A	0.03	0.23	0.28	**0.40***	0.22	**-0.29***	-0.17	-0.15	**0.61***	**0.50***	**0.69***	**0.70***	-0.10	-0.22	0.12	-	0.75
HADS (A+D)	0.06	0.12	0.23	**0.43***	**0.32***	-0.25	-0.10	-0.06	**0.50***	**0.42***	**0.54***	**0.57***	-0.20	-0.25	0.70	0.75	-

Notes: *- significant correlation, 6MWT: the 6-minute walk test, FEV1%: forced expiratory volume for 1 second, BMI: Body Mass Index, MET: metabolic equivalent, HADS: Hospital Anxiety and Depression Scale
